# Generalizability of Blood Pressure Lowering Trials to Older Patients: Cross-Sectional Analysis

**DOI:** 10.1111/jgs.16749

**Published:** 2020-09-08

**Authors:** James P. Sheppard, Mark Lown, Jenni Burt, Eleanor Temple, Rebecca Lowe, Hannah Ashby, Oliver Todd, Julie Allen, Gary A. Ford, Rosalyn Fraser, Carl Heneghan, F.D. Richard Hobbs, Sue Jowett, Paul Little, Jonathan Mant, Jill Mollison, Rupert Payne, Marney Williams, Ly-Mee Yu, Richard J. McManus

**Affiliations:** * Nuffield Department of Primary Care Health Sciences, University of Oxford, Oxford, UK; † Primary Care Population Sciences and Medical Education Unit, Faculty of Medicine, University of Southampton, Southampton, UK; ‡ The Healthcare Improvement Studies Institute, University of Cambridge, Cambridge, UK; § Academic Unit of Elderly Care and Rehabilitation, University of Leeds , Leeds, UK; ¶ Radcliffe Department of Medicine, University of Oxford, Oxford, UK; ∥ Institute of Applied Health Research, University of Birmingham, Birmingham, UK; ** Primary Care Unit, Department of Public Health and Primary Care, University of Cambridge, Cambridge, UK; †† Centre for Academic Primary Care, University of Bristol, Bristol, UK; ‡‡ Patient and Public Involvement Representative, London, UK

**Keywords:** hypertension, cardiovascular disease, frailty, electronic health records, randomized controlled trials

## Abstract

**Background/Objectives:**

Randomized controlled trials are used to inform clinical guidelines on the management of hypertension in older adults, but it is unclear to what extent these trials represent the general population attending routine clinical practice. This study aimed to define the proportion and characteristics of patients eligible for hypertension trials conducted in older people.

**Design:**

Cross-sectional study.

**Setting:**

A total of 24 general practices in England.

**Participants:**

Anonymized electronic health record data from all individuals aged 80 and older.

**Measurements:**

Descriptive statistics were used to define the proportion and characteristics of patients eligible for two previous medication intensification trials (HYVET, SPRINT) and one medication reduction trial (OPTiMISE). A logistic regression model was constructed to estimate predictors of eligibility for each trial.

**Results:**

Of 15,376 patients identified, 268 (1.7%; 95% confidence interval [CI] = 1.5–2.0%), 5,290 (34.4%; 95%CI = 33.7–35.2%), and 3,940 (25.6%; 95%CI = 24.9–26.3%) were eligible for the HYVET, SPRINT, and OPTiMISE trials, respectively. Between 5.6% and 30.7% of exclusions from each trial were due to eligibility criteria excluding those with high or uncontrolled blood pressure. Frailty (odds ratio [OR] = .44; 95%CI = .36–.54 [OPTiMISE]), cardiovascular polypharmacy (OR = .61; 95% CI = .55–.68 [SPRINT]) and multimorbidity (OR = .72; 95%CI = .64–.82 [SPRINT]) were associated with a lower likelihood of being eligible for one or more of the trials.

**Conclusion:**

A possible unintended consequence of blood pressure criteria used by trials attempting to answer different primary questions is that for many older patients, no trial evidence exists to inform treatment decisions in routine practice. Caution should be exercised when applying results from existing trials to patients with frailty or multimorbidity.

Hypertension is a major modifiable risk factor for cardiovascular disease (CVD),^1^ and an individual’s risk of a cardiovascular event can be significantly reduced with treatment to lower blood pressure.^2^ In older individuals aged 80 and older, previous trials showed benefit with treatment,^3,4^ although meta-analyses of these and other trials indicated that larger blood pressure reductions and multiple antihypertensive prescriptions may also be harmful.^5,6^ Observational studies also suggested that blood pressure lowering in older patients may be harmful, particularly in patients with frailty and multimorbidity, increasing the risk of falls,^7^ acute kidney injury,^8^ and death.^9,10^

The two largest trials that have examined the efficacy of antihypertensive treatment in patients aged 80 and older are the Hypertension in the Very Elderly Trial (HYVET)^3^ and the Systolic Blood Pressure Intervention Trial (SPRINT).^4^ HYVET showed in patients with a baseline systolic blood pressure of 160 mm Hg and higher that blood pressure lowering to a target of 150 mm Hg systolic reduces the risk of CVD, heart failure, and death from any cause.^3^ The SPRINT trial showed that in patients with a baseline systolic blood pressure of 130 mm Hg and higher, systolic blood pressure lowering to 120 mm Hg (defined using automated and mostly unaccompanied measurement methods) reduced the risk of CVD and death,^4^ and this effect was sustained in a subgroup of participants aged 80 and older, although not in those with reduced cognitive function.^11^

However, it is unclear to what extent these results apply to older patients in routine practice,^12,13^ particularly with population-wide observational data suggesting treatment may be harmful.^7–9^ Indeed, patients in the HYVET trial are thought to have had fewer comorbidities,^14^ whereas it is unclear to what extent the levels of frailty observed in the SPRINT population^15^ compared with those in the general populations of Europe and North America.^16,17^

The 2018 Optimizing Treatment for Mild Systolic Hypertension in the Elderly (OPTiMISE) trial examined the safety of reducing blood pressure medications in older patients with controlled systolic blood pressure (<150 mm Hg).^18^ The rationale for OPTiMISE was that patients with frailty and multimorbidity are not well represented in previous blood pressure lowering trials and may in fact benefit from fewer treatments, due to a reduction in adverse events from polypharmacy.^19,20^ The present study aimed to examine this rationale by (1) determining the proportion of patients registered at a primary care practice who would be eligible for each of the HYVET, SPRINT, and OPTiMISE trials; (2) establishing what patient characteristics are associated with being eligible, and (3) comparing the characteristics of patients in the participating practices who would have been eligible for the trials with those actually enrolled in each trial.

## Methods

Detailed methods are given in the Supplementary Methods S1 (Supplementary Appendix).

### Study Design

This study used a cross-sectional design, utilizing anonymized data extracted from the medical records of patients registered at general practices enrolled in the OPTiMISE trial.^18^ Data were extracted between September 2017 and October 2018 using Egton Medical Information Systems (EMIS) search and reporting software (Egton Medical Information Systems Health, Leeds, UK). The study was approved by a National Health Service Research Ethics Committee (South Central-Oxford A; ref 16/SC/0628). Because data were fully anonymized, no individual patient consent was required.

### Population

Eligible patient data were those from individuals aged 80 and older and registered at English general practices hosting the OPTiMISE trial. General practices had to be using the EMIS electronic health record system and agree to anonymized patient data being extracted for the study.

### Study Outcomes

The primary outcome for the study was the proportion of patients in the participating practices who would have been eligible for the HYVET, SPRINT, and OPTiMISE trials. These trials were chosen because they represent the largest trials conducted to date examining hypertension management strategies (both prescribing and deprescribing) in older adults. Secondary outcomes were to describe the characteristics of eligible patients, to determine the proportion of individuals excluded by each eligibility criteria, and to identify independent predictors of eligibility. The characteristics of those eligible in the participating practices were compared with those enrolled in each trial. Patients were classified as eligible if they fulfilled the published inclusion and exclusion criteria for each trial (Table 1). Due to the use of data from electronic health records, it was not possible to apply some eligibility criteria to the sample population (Supplementary Table S1).

### Covariates

Data relating to baseline patient characteristics (age, sex, smoking status, and body mass index [BMI]), blood pressure, cholesterol, comorbidities, and details of all prescribed cardiovascular medications were extracted. A frailty index (FI) was developed retrospectively from 26 available deficits. Frailty was categorized using the thresholds reported in the SPRINT trial: fit (FI <.1), less fit (FI .1–.21), and frail (FI >.21). Cardiovascular polypharmacy was defined as being prescribed three or more medications for cardiovascular conditions (eg, antihypertensive, statin, antiplatelet, or diabetic medications).

To examine predictors of eligibility for each trial, missing data for blood pressure and BMI were assumed missing at random and imputed using multiple imputation with chained equations.^21^ Data on the characteristics of patients actually enrolled in each trial were extracted from previous publications (HYVET, SPRINT)^3,11^ or obtained from the original trial data set (OPTiMISE).^18^

### Main Analyses

Descriptive statistics were used to define the primary outcome, the characteristics of eligible patients, and the proportion of patients excluded by each eligibility criteria. To better understand the phenotype of patients eligible for each trial, a logistic regression model was constructed to examine predictors of eligibility for each trial, with general practice (site) included as a random effect. Candidate predictors for each model were prespecified as age, sex, blood pressure, BMI, frailty, number of comorbidities, number of cardiovascular medications, history of hypertension, chronic kidney disease, diabetes mellitus, type II, myocardial infarction, stroke (or transient ischemic attack), and heart failure or coronary artery bypass graft. Comparisons between the characteristics of eligible patients and those included in the original trials were made using independent samples *t* tests and two sample tests of proportions. Bonferroni correction was used to account for multiple comparisons.

### Sensitivity Analyses

The definition of clinic blood pressure (most recently recorded reading) was examined in sensitivity analyses where the blood pressure eligibility criteria for each trial were defined according to the mean of the last three readings documented in the medical records. Further analyses examined the proportion of patients with hypertension (defined by a coded diagnosis or prescription of antihypertensive therapy) who would have been eligible for each trial. For logistic regression models examining predictors of eligibility, sensitivity analyses were undertaken based on complete cases only, to establish the impact of using multiple imputations to deal with missing data. Further sensitivity analyses were undertaken on complete cases using a standard logistic regression model with backward stepwise selection of candidate predictors, with the significance level for inclusion set to *P* < .05. All analyses were conducted using STATA v.14.1 (Special Edition, StataCorp, College Station, TX).

## Results

### Primary Results

**Figure 1:**
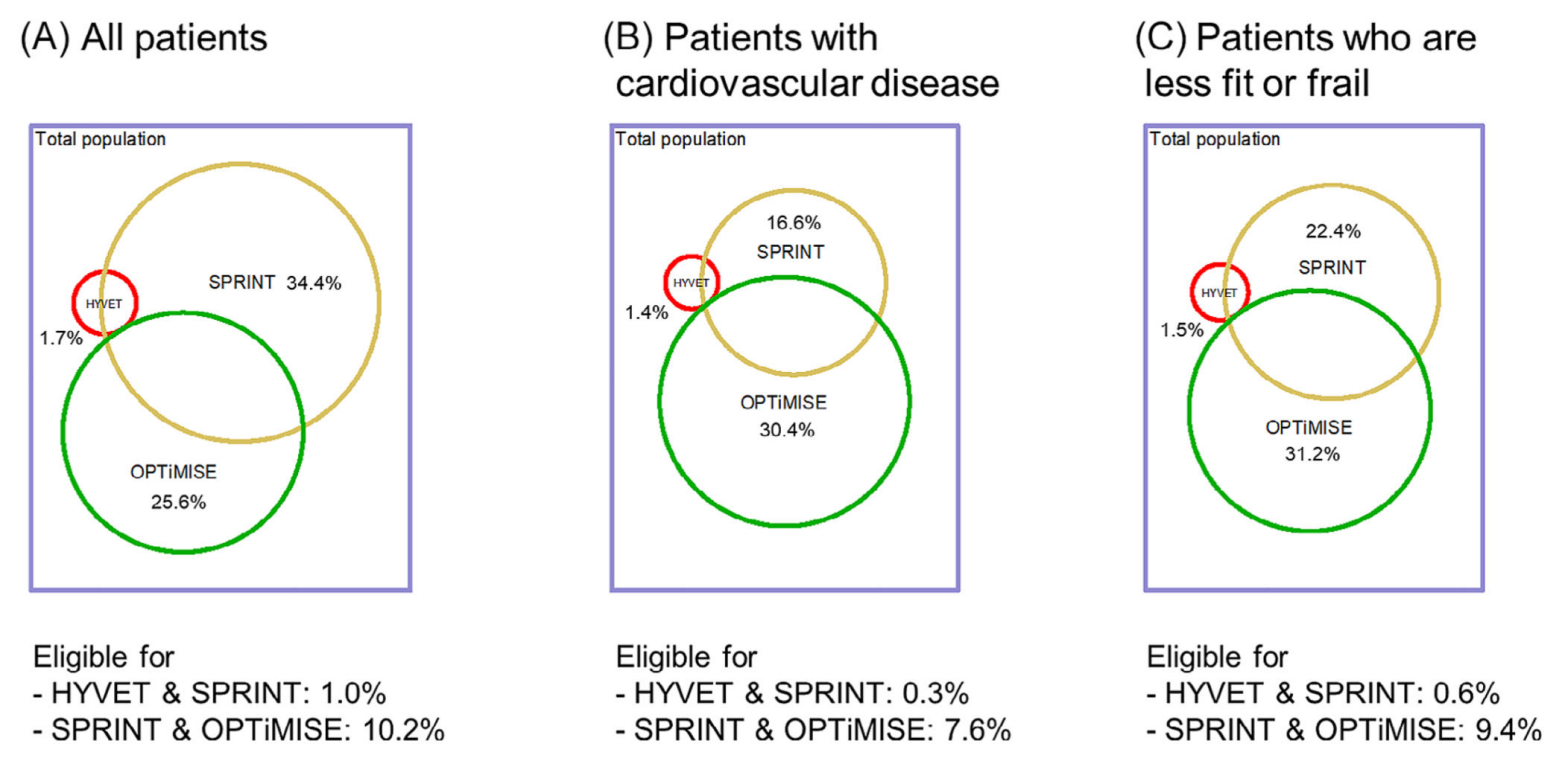
Proportion of patients eligible for each trial in the total population, those with cardiovascular disease and frailty. [Color figure can be viewed at wileyonlinelibrary.com]

**Figure 2:**
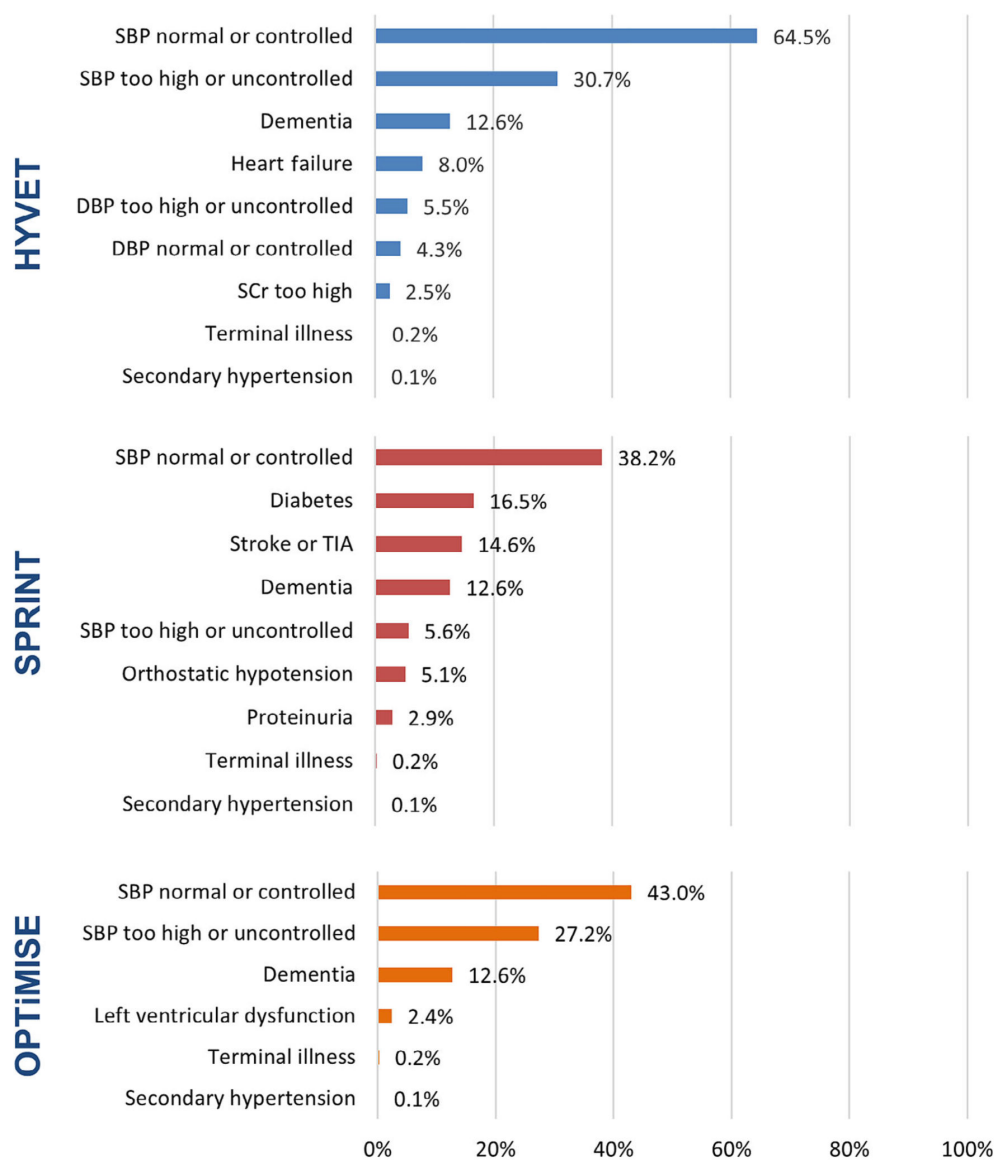
Proportion of patients excluded by each eligibility criteria (n = 15,376). DBP, diastolic blood pressure; SBP, systolic blood pressure; SCr, serum creatinine; TIA, transient ischemic attack.

Overall, 24 of 69 general practices participating in the OPTiMISE trial were using EMIS Health software and agreed to provide data for this study. A total of 15,376 patients aged 80 and older were registered at these practices. Approximately 268 (1.7%; 95% confidence interval [CI] = 1.5–2.0%), 5,290 (34.4%; 95% CI = 33.7–35.2%), and 3,940 (25.6%; 95% CI = 24.9–26.3%) patients would have been eligible for the HYVET, SPRINT, and OPTiMISE trials, respectively (Figure 1). No patients were eligible for both HYVET and OPTiMISE trials due to mutually exclusive blood pressure eligibility criteria, but there was some overlap in eligibility for HYVET and SPRINT, and SPRINT and OPTiMISE (Figure 1). Having normal or controlled systolic blood pressure was the most common reason for exclusion from each trial, but up to one-third of patients with high or uncontrolled blood pressure were also ineligible (HYVET = 30.7%; SPRINT = 5.6%; OPTiMISE = 27.2%). Diagnosis of dementia (12.6%), diabetes mellitus, type II (16.5% [SPRINT only]), or stroke (14.6% [SPRINT only]) were other common reasons for exclusion (Figure 2).

Sensitivity analyses examining blood pressure defined as the mean of the last three recorded readings identified similar proportions of patients eligible for each trial (data available from authors). Similar proportions of patients were eligible for each trial when the population was limited to those with a previous diagnosis of hypertension, with the exception of OPTiMISE that had more eligible hypertensive patients (35.0%; 95% CI = 34.1–35.9%).

### Characteristics of Eligible Patients

Patients eligible for each trial were similar in age and BMI, but they had different levels of mean systolic blood pressure (169 ± 9 mm Hg [HYVET]; 141 ± 10 mm Hg [SPRINT]; 131 ± 12 [OPTiMISE]; 132 ± 16 mm Hg [general population]), reflecting the different entry criteria. Patients eligible for the HYVET and SPRINT trials had less frailty and multimorbidity than those eligible for OPTiMISE (Table 2 and Supplementary Table S3). The proportion of cardiovascular morbidities varied across trials, and the mean number of cardiovascular medications was highest in patients eligible for the OPTiMISE trial (Table 2 and Supplementary Table S3).

### Predictors of Eligibility

**Figure 3:**
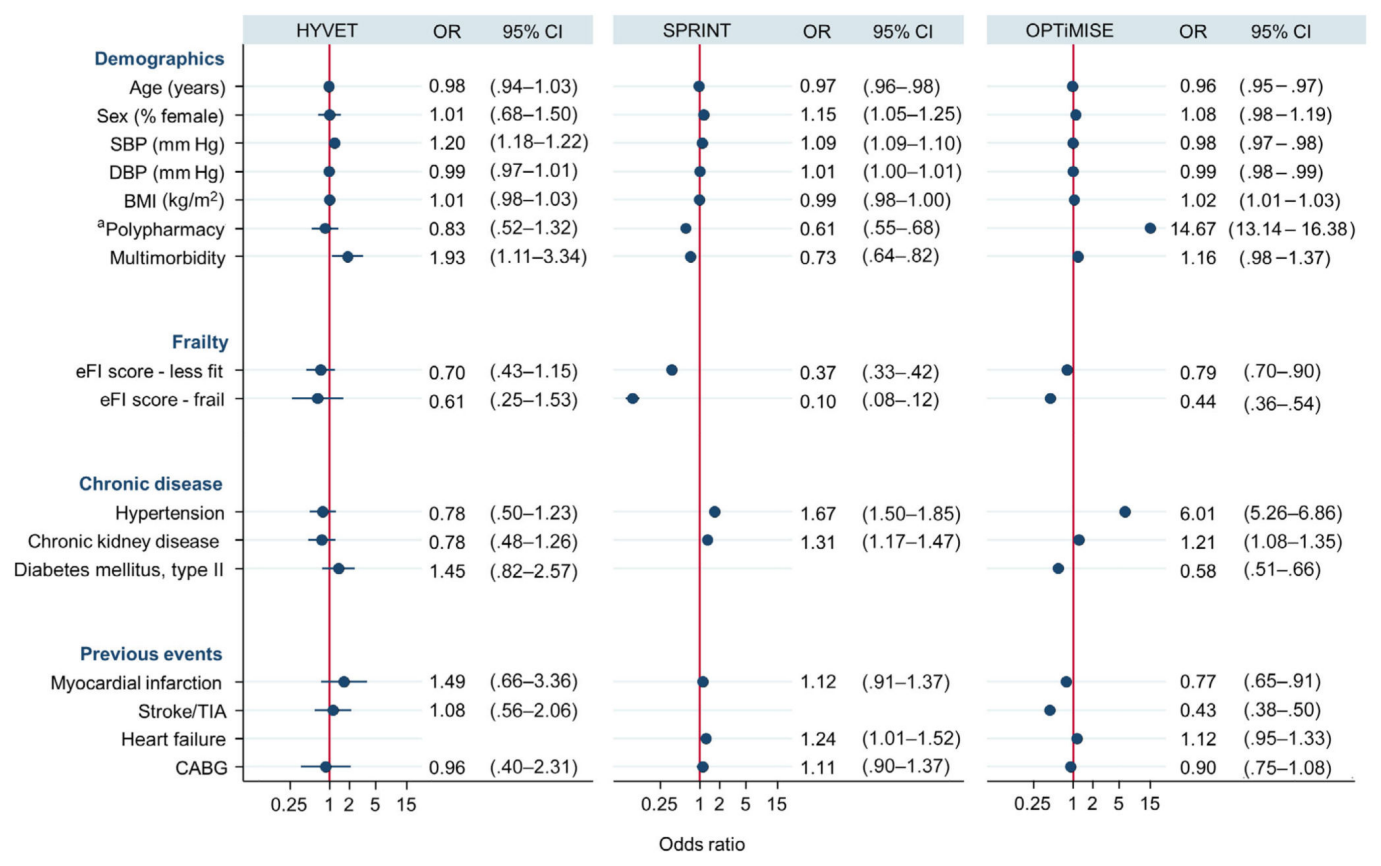
Coefficient plot showing results of logistic regression analysis examining predictors of eligibility for each trial (n = 15,376). ^a^Polypharmacy defined as being prescribed three or more cardiovascular medications. BMI, body mass index; CABG, coronary artery bypass graft; CI, confidence interval; DBP, diastolic blood pressure; eFI, electronic frailty index; OR, odds ratio; SBP, systolic blood pressure; TIA, transient ischemic attack. [Color figure can be viewed at wileyonlinelibrary.com]

Patients with higher systolic blood pressure were more likely to be eligible for the HYVET and SPRINT trials (odds ratio [OR] = 1.20; 95% CI = 1.18–1.22 [HYVET]; OR = 1.09; 95% CI = 1.09–1.10 [SPRINT]) but less likely to be eligible for OPTiMISE (OR = .98; 95% CI = .97–.98), again reflecting the opposing eligibility criteria for these trials (Figure 3). Having frailty was associated with a lower likelihood of being eligible for all trials (OR = .66; 95% CI = .53–1.37 [HYVET]; OR = .09; 95% CI = .08–.12 [SPRINT]; and OR = .44; 95% CI = .36–.54 [OPTiMISE]). The presence of cardiovascular polypharmacy and multimorbidity were associated with lower odds of being eligible for the SPRINT trial (OR = .61; 95% CI = .55–.68 [polypharmacy]; OR = .72; 95% CI = .64–.82 [multimorbidity]) but higher odds of being eligible for the OPTiMISE trial (OR = 14.67; 95% CI = 3.14–16.38 [polypharmacy]; OR = 1.16; 95% CI = .97–1.37 [multimorbidity]). Results were similar in sensitivity analyses examining complete cases only (Supplementary Figure S1). Analyses using backward stepwise selection identified systolic blood pressure and reduced frailty as factors predicting eligibility across all three trials (Supplementary Figure S2).

### Eligible Patients Compared with Recruited Patients

Significant differences were found between those deemed eligible in the present study and those recruited to each individual trial (Supplementary Table S3). Patients enrolled in the HYVET trial were on average younger, with higher blood pressure, lower BMI, and fewer cardiovascular morbidities compared with the eligible population in the practices considered (*P* < .002). Patients in the SPRINT trial were also younger, had lower diastolic blood pressure but higher systolic blood pressure, BMI, and more cardiovascular morbidities and treatment (*P* < .001). Participants in the OPTiMISE trial had lower diastolic blood pressure, and fewer had a history of stroke, CVD, or prescriptions for statin and antiplatelet therapy than the total population that would have been eligible (*P* < .001; Supplementary Table S3).

## Discussion

### Summary of Main Findings

This study examined the characteristics of 15,376 patients aged 80 and older registered in 24 general practices in England. We found that most patients would not have been eligible for randomized controlled trials that inform clinical guidelines^22–25^ on the management of hypertension. Most of the ineligibility related to the differing blood pressure eligibility criteria used by trials attempting to answer different primary questions, with up to a one-third of individuals with high or uncontrolled blood pressure excluded. This is perhaps not surprising. However, a possible unintended consequence of these criteria was that eligible patients were less likely to be frail and more likely to be prescribed multiple cardiovascular medications or have multimorbidity. Therefore, for many older patients, no randomized controlled trials exist to directly inform treatment decisions in routine practice.

### Strengths and Weaknesses

This study examined data from a large sample of more than 15,000 patients registered to general practices across the south-central region of England. Individuals were representative of the region and country in terms of age but may not be reflective of practices in other parts of the country that serve populations differing in ethnicity and social deprivation.^26^ It is also possible that some selection bias may have been present because only 24 of 69 practices approached were able to participate.

The eligibility criteria that accounted for the largest number of exclusions were those related to having high or low systolic blood pressure. Our definition of systolic blood pressure, based on the most recently recorded value, almost certainly differs from the standardized readings taken in the original trials.^27^ However, readings reflected routine practice, and the same definition was used for each trial, and so comparisons between trials are still likely to be valid.

Our analyses were unable to consider the proportion of eligible individuals who would likely give informed consent to participate in each trial or the proportion deemed in clinical equipoise by the treating physician. Indeed, in the OPTiMISE trial, as few as 9% of those deemed potentially eligible on the basis of information held in electronic health records gave informed consent and were randomized, suggesting that these estimates are likely to be conservative. Data presented in Table 2 suggest the decision to participate may have been influenced by patient characteristics such as sex, frailty, multimorbidity, and treatment prescription. It was not possible to examine the association between ethnicity and trial eligibility due to large amounts of missing data in the electronic health records for this patient characteristic.

Derivation of the FI was post hoc, and therefore some of the deficits described in previous models were not extracted from individual electronic health records.^28^ Of the 26 deficits available, each of the five domains that make up a valid cumulative deficit frailty model were covered: signs, symptoms, disease, disability, and abnormal laboratory results.^29^ We therefore consider our frailty model to be valid. Models used to define frailty in each of the previous trials examined here were also derived post hoc and included a wide variety of deficits (Supplementary Table S2).^15,28,30^ The current analysis offered a unique opportunity to estimate frailty and compare across trial-eligible populations using a consistent definition.

### Comparison with Previous Literature

A study from the Netherlands found that in a population of patients attending a geriatric day clinic, less than one-half would have been eligible for the HYVET trial, and those eligible would have had significantly fewer comorbidities.^14^ The present study found of those older patients attending routine primary care, fewer than 2% fulfilled the eligibility criteria for HYVET, although those that were eligible had comparable multimorbidity. Furthermore, our finding that 34.4% of older patients would have been eligible for the SPRINT trial was very similar to that of Bress et al.,^31^ who examined patients from the National Health and Nutrition Examination Survey database and found 34.6% of patients aged 75 and older would have been eligible for the trial.

The present study found that eligible populations differed significantly from those who participated in the original trials. This accentuated the differences between eligible patients and the general population. Participating patients had higher blood pressure and BMI but fewer comorbidities.

### Implications for Clinical Practices

Clinical guidelines for the management of hypertension in older patients continue to recommend lower thresholds for treatment initiation and targets for patients already on medication.^22,24,25^ Most suggest exercising clinical judgment in patients with multimorbidity and frailty,^23,24^ and some go as far as to explicitly cite the exclusion criteria from the SPRINT trial in reference to patients where such a strategy may not be appropriate.^25^ The present data show the extent to which clinical judgment may be required with as many as two-thirds of patients aged older than 80 not meeting the eligibility criteria for previous trials.

Further work is needed to better understand if differences between recruited trial participants and the general population are important in determining the safety and efficacy of treatments. Ultimately, it is not feasible to conduct randomized controlled trials in all populations, but attempts should be made to better capture patients from underrepresented groups (such as those with frailty and multimorbidity) in future trials. One approach might be to consider the use of specific inclusion/exclusion criteria designed to maximize the number of patients with frailty and multimorbidity eligible.

In conclusion, the present study found that most patients aged older than 80 would not have been eligible for large randomized controlled trials examining hypertension management strategies in older adults, albeit mainly due to differing blood pressure control thresholds. Therefore, for many older patients, no randomized controlled trials exist that directly inform treatment decisions in routine practice, and so caution should be exercised when using existing data in older patients with frailty or multimorbidity.

## Supplementary Material

Additional Supporting Information may be found in the online version of this article.

**Supplementary Appendix S1:** Supporting Information.

Supporting Information

## Figures and Tables

**Table 1:** Eligibility Criteria for Each Trial^a^

Covariate	Eligibility criteria applied		
	HYVET trial	SPRINT trial (older subgroup)	OPTiMISE trial
Age, y	≥80	≥80	≥80
Sex	Male and female	Male and female	Male and female
Systolic blood pressure	160–199 mmHg	130–180 mm Hg (0–1 meds), or 130–170 mm Hg (2 meds), or 130–160 mm Hg (3 meds), or 130–150 mm Hg (4 meds)	<150 mm Hg (≥2 meds)
Diastolic blood pressure	<110 mm Hg		
Serum creatinine	<150 μmoL/L		
Dementia, secondary hypertension, terminal illness	Exclude	Exclude	Exclude
Angina, IHD, ACS, VHD		Exclude (if occurred in past 3 mo)	
Stroke or TIA		Exclude	Exclude (if occurred in past 12 mo)
Diabetes mellitus, type II, proteinuria, PKD, glomerulonephritis, organ transplant		Exclude	
Heart failure	Exclude (congestive heart failure with a diuretic or ACEi)	Exclude (if occurred in past 6 mo)	
Malignant hypertension	Exclude		Exclude
Left ventricular dysfunction			Exclude (if only on ACEis/ARBs and/or beta-blockers and/or spironolactone)
Myocardial infarction		Exclude (if occurred in past 3 mo)	Exclude (if occurred in past 12 mo)
Orthostatic hypotension		Exclude (standing SBP <110 mm Hg)	
Alcohol or drug abuse	Exclude	Exclude (if occurred in past 12 mo)	
In nursing home	Exclude	Exclude	
Unintentional weight loss		Exclude (if occurred in past 6 mo)	

Abbreviations: ACEi, angiotensin-converting enzyme inhibitor; ACS, acute coronary syndrome; ARB, angiotensin receptor blocker; HYVET, Hypertension in the Very Elderly Trial; IHD, ischemic heart disease; OPTiMISE, Optimizing Treatment for Mild Systolic Hypertension in the Elderly [trial]; PKD, polycystic kidney disease; SBP, systolic blood pressure; SPRINT, Systolic Blood Pressure Intervention Trial; TIA, transient ischemic attack; VHD, valvular heart disease.

a Applied to data from electronic health records in this analysis.

**Table 2:** Characteristics of Patients Eligible and Enrolled to Each Trial

Characteristic	HYVET trial				SPRINT trial				OPTiMISE trial		
	Eligible population	Reported population^3^	*P* value^a^		Eligible population	Reported population^11^	*P* value^a^		Eligible population	Reported population^18^	*P* value^a^
Patient characteristics											
Total population	268	3,845	—		5,290	1,167	—		3,940	569	—
Age, y	85.6	83.6	< .001		85.0	83.5	< .001		84.8	84.8	1.000
Sex, % female	67.2	60.5	.097		61.5	38.7	< .001		58.0	51.5	.003
SBP, mm Hg	169.3	173.0	< .001		141.2	142.5	< .001		130.8	130.0	.134
DBP, mm Hg	81.2	90.8	< .001		75.6	70.0	< .001		71	69.3	< .001
BMI, kg/m^2^	26.6	24.7	< .001		26.4	27.1	< .001		27.7	27.6	.725
Frailty score: fit, %	50.8	—	—		63.2	—	—		31.0	22.3	< .001
Frailty score: less fit, %	42.2	—	—		33.9	—	—		55.3	59.9	.038
Frailty score: frail, %	7.1	—	—		2.9	—	—		13.7	17.8	.008
Electronic frailty index, median	.09	.16	—		.09	—	—		.13	.14	—
Medical history, %											
Hypertension	65.7	89.9	< .001		59.8	—	—		89.4	96.1	< .001
Myocardial infarction	8.2	3.1	< .001		4.4	—	—		10.1	7.0	.019
Stroke/TIA	11.9	6.8	.001		.0	.0	—		13.0	7.9	< .001
CVD	28.0	11.8	< .001		17.3	27.2	< .001		42.4	30.4	< .001
Diabetes mellitus, type II	14.6	6.8	< .001		.0	.0	—		23.0	17.8	.005
CKD	25.0	—	—		22.5	50.6	< .001		36.8	32.7	.057
Prescribed medications, %											
Antihypertensive	62.7	64.7	.508		61.4	—	—		100.0	100.0	1.00
Statin	33.6	—	—		32.1	50.7	< .001		61.0	33.2	< .001
Antiplatelet	25.4	—	—		15.8	60.2	< .001		31.8	19.5	< .001

Abbreviations: BMI, body mass index; CKD, chronic kidney disease; CVD, cardiovascular disease; DBP, diastolic blood pressure; HYVET, Hypertension in the Very Elderly Trial; OPTiMISE, Optimizing Treatment for Mild Systolic Hypertension in the Elderly [trial]; SBP, systolic blood pressure; SPRINT, Systolic Blood Pressure Intervention Trial; TIA, transient ischemic attack.

a Data relating to the reported populations were extracted from previous publications (HYVET, SPRINT)^3,11^ or obtained from the original trial data set (OPTiMISE).^18^ Comparisons were made using independent samples *t* tests and two sample tests of proportions. Standard deviations required for this analysis are taken from the variance reported in the intervention arms of the original HYVET and SPRINT trials, giving conservative estimates of the difference between groups. Bonferoni correction for significance level was *P* < .002.
