# Supplementary material for: Generalizability of Blood Pressure Lowering Trials to Older Patients: Cross-Sectional Analysis
Source: J Am Geriatr Soc. Author manuscript; Available in PMC 2025 Sep 22. (PMC7618156; doi:10.1111/jgs.16749)
Supplement: Supporting Information [file EMS208802-supplement-Supporting_Information.pdf]

Generalisability of blood pressure lowering trials to older patients: cross-sectional analysis

Supplemental material

James Sheppard,[1] Mark Lown,[2] Jenni Burt,[3] Eleanor Temple,[1] Rebecca Lowe,[1] Hannah Ashby,[1] Oliver Todd,[4] Julie Allen,[1] Gary Ford,[5] Rosalyn Fraser,[1] Carl Heneghan,[1] FD Richard Hobbs,[1] Sue Jowett,[6] Paul Little,[2] Jonathan Mant,[7] Jill Mollison,[1] Rupert Payne,[8] Marney Williams,[9] Ly-Mee Yu,[1] and Richard J McManus[1]

[1]Nuffield Department of Primary Care Health Sciences, University of Oxford, Oxford, UK
[2]Primary Care Population Sciences and Medical Education Unit, Faculty of Medicine, University of Southampton, Southampton, UK
[3]The Healthcare Improvement Studies Institute, University of Cambridge, Cambridge, UK
[4]Academic Unit of Elderly Care and Rehabilitation, University of Leeds, UK
[5]Radcliffe Department of Medicine, University of Oxford, UK
[6]Institute of Applied Health Research, University of Birmingham, Birmingham, UK
[7]Primary Care Unit, Department of Public Health and Primary Care, University of Cambridge, Cambridge, UK
[8]Centre for Academic Primary Care, University of Bristol, Bristol, UK
[9]Patient and public involvement representative, London, UK

Contents

1. **Supplemental Methods S1**
2. **Table S1.** Eligibility criteria which could not be applied to available routine electronic health record data
3. **Table S2.** Frailty models used in each trial
4. **Table S3.** Characteristics of patients eligible for each trial
5. **Figure S1.** Sensitivity analysis: Coefficient plot showing results of logistic regression analysis examining predictors of eligibility for each trial (complete cases only)
6. **Figure S2.** Sensitivity analysis: Coefficient plot showing results of logistic regression analysis with backwards stepwise selection of candidate predictors of eligibility for each trial (complete cases only)

Supplemental Methods S1

Study design

This study used a cross-sectional design, utilising anonymised data extracted from the medical records of patients registered at general practices enrolled onto the OPTiMISE trial.(24) Data were extracted between September 2017 and October 2018 using EMIS search and reporting software (Egton Medical Information Systems Health, Leeds, UK) embedded into the practice electronic health record system. The study was approved by an NHS Research Ethics Committee (South Central - Oxford A; ref 16/SC/0628). Since data were fully anonymised, no individual patient consent was required.

Population

Eligible patient data were those from individuals aged ≥80 years and registered at English general practices hosting the OPTiMISE trial. General practices had to be using the EMIS Health electronic health record system and agree to anonymised patient data being extracted for the study.

Study outcomes

The primary outcome for the study was the proportion of patients in the participating practices who would have been eligible for the HYVET, SPRINT and OPTiMISE trials. These trials were chosen because they represent the largest trials conducted to date examining hypertension management strategies (both prescribing and deprescribing) in older adults. Secondary outcomes were to describe the characteristics of eligible patients, the proportion of individuals excluded by each eligibility criteria and to identify independent predictors of eligibility. The characteristics of those eligible in the participating practices were compared to those enrolled onto each trial. Patients were classified as being eligible if they fulfilled the published inclusion and exclusion criteria for each trial (table 1). Due to the use of data from electronic health records, it was not possible to apply some eligibility criteria to the sample population. For example, potentially eligible patients for the HYVET trial were excluded if they were unable to stand, had participated in a drug trial in the preceding month or the investigator deemed them to have contraindications to the study medications and these criteria could not be determined from the data available in this study. A full list of eligibility criteria which could not be applied to the present data are given in Table S1.

Covariates

Data relating to baseline patient characteristics (age, sex, smoking status and body mass index [BMI]), blood pressure, cholesterol, co-morbidities and details of all prescribed cardiovascular medications were extracted. Continuous data were cleaned and outlying values excluded where appropriate. Blood pressure was defined by the most recent clinic reading documented in the electronic health records. Blood pressure outliers were defined as a systolic blood pressure of <70mmHg or >260mmHg and a diastolic blood pressure of <40mmHg or >150mmHg as previously described.(27) BMI values were excluded if they were documented as <10 kg/m^2 or >100 kg/m^2 (based on expert opinion). Values for LDL cholesterol (>10 mmol/L), HDL cholesterol (<1 or >4.7mmol/L), serum creatinine (<10 or >300 µmol/L) and transaminase (<10 or >100 µmol/L) were excluded according to previously published plausible ranges or being outliers from the normal distribution of data.(28, 29)

Smoking status was defined as never, previous, or current smoker. Those with unknown smoking status were assumed to have never smoked. A frailty index (FI) was developed retrospectively (as was done in HYVET and SPRINT), with a total of 26 deficits available to estimate a score.(16, 30) These deficits covered each of the five domains which contribute to the cumulative deficit frailty model: signs, symptoms, disease,

disability and abnormal laboratory results.(31) Frailty was categorised using the thresholds reported in the SPRINT trial: fit (FI<0.1), less fit (FI 0.1 to 0.21), frail (FI>0.21).

Where there was no record of blood pressure lowering, statin or antiplatelet treatment, or co-morbidities, it was assumed that patients were not prescribed treatment or the co-morbidity was not present. Cardiovascular polypharmacy was defined as being prescribed three or more medications for cardiovascular conditions (e.g. antihypertensive, statin, antiplatelet or diabetic medications).

To examine predictors of eligibility for each trial, missing data for blood pressure and BMI were assumed missing at random and imputed using multiple imputation with chained equations.(32) Separate imputation models were created for each outcome (HYVET eligibility, SPRINT eligibility and OPTiMISE eligibility) and included all covariates examined in the main analysis and the outcome of interest.(33) Each model was based on 20 imputations. Data on the characteristics of patients actually enrolled in each trial were extracted from previous publications (HYVET, SPRINT)(3, 18) or obtained from the original trial dataset (OPTiMISE).(24)

Main analyses

Descriptive statistics were used to define the primary outcome, the characteristics of eligible patients, and the proportion of patients excluded by each eligibility criteria. Venn diagrams were used to display the overlap in proportion of patients eligible for each trial, including in those with previous cardiovascular disease or who were less fit and frail. To better understand the phenotype of patients eligible for each trial, a logistic regression model was constructed to examine predictors of eligibility for each trial, with general practice (site) included as a random effect. Candidate predictors for each model were pre-specified as age, sex, blood pressure BMI, frailty, number of co-morbidities, number of cardiovascular medications, history of hypertension, chronic kidney disease, diabetes, myocardial infarction, stroke (or transient ischemic attack), heart failure or coronary artery bypass graft. These included age, sex, blood pressure, BMI, chronic disease status, history of cardiovascular disease, cardiovascular polypharmacy and frailty. Due to the statistical model used and the need for multiple imputation to deal with missing data, candidate predictor selection methods were not used to reduce the model.

Comparisons between the characteristics of eligible patients and those included in the original trials were made using independent samples t-tests and two sample tests of proportions. Bonferroni correction was used to account for multiple comparisons.

Sensitivity analyses

The definition of clinic blood pressure (most recently recorded reading) was examined in sensitivity analyses where the blood pressure eligibility criteria for each trial were defined according to the mean of the last three readings documented in the medical records. Further analyses examined the proportion of patients with hypertension (defined by a coded diagnosis or prescription of antihypertensive therapy) who would have been eligible for each trial. For logistic regression models examining predictors of eligibility, sensitivity analyses were undertaken based on complete cases only, to establish the impact of using multiple imputations to deal with missing data in the primary analysis. Further sensitivity analyses were undertaken on complete cases using a standard logistic regression model with backward stepwise selection of candidate predictors, with the significance level for inclusion set to $p<0.05$.

All analyses were conducted using STATA version 14.1 (Special Edition, StataCorp, Texas, USA). Results are presented as means, medians or proportions, with standard deviations or 95% confidence intervals, unless otherwise stated.

Table S1. Eligibility criteria for each trial (which could not be applied to data from electronic health records in this analysis)

HYVET	SPRINT	OPTiMISE
Unable to stand	Indication for specific BP lowering meds/intolerant to specific meds	In the Investigator's opinion, could potentially benefit from medication reduction due to existing polypharmacy, co-morbidity, non-adherence or dislike of medicines and/or frailty
Participating in a drug trial in preceding month	Arm circumference too large or small	A participant has heart failure but has not had an echocardiogram since its onset
Contraindications to medications	Factors judged by the clinic team to be likely to limit adherence to interventions	Investigator deems that there is a compelling indication for medication continuation
Gout (missing variable)	A medical condition likely to limit survival to less than 3 years, or a cancer diagnosed and treated within the past 2 years that, in the judgment of clinical study staff, would compromise a participant's ability to comply with the protocol	Participants who have participated in another research trial involving antihypertensive medication in the past 4 weeks
	Participating in another clinical trial	
	Living in the same household as an already randomized SPRINT participant	

Table S2. Cumulative deficit frailty models used in each study

HYVET	SPRINT	OPTiMISE	Present study
Low Haematocrit		Anaemia and haematinic deficiency	Anaemia and haematinic deficiency
		Arthritis	Arthritis
Atrial fibrillation	Self-reported history of Atrial fibrillation	Atrial fibrillation	Atrial fibrillation
	Self-reported history of cancer		Cancer
	Self-reported history of stroke	Cerebrovascular disease	Cerebrovascular disease
	Chronic kidney disease	Chronic kidney disease	Chronic kidney disease
	Reduced Global Cognitive Status (MoCA)	Memory and cognitive problems	Dementia
Diabetes	Self-reported history of diabetes	Diabetes	Diabetes
		Fragility fracture	Fragility fracture
	Self-reported history of heart failure	Heart failure	Heart failure
		Hypertension	Hypertension
		Dizziness	Hypoglycemic coma
Difficulty bathing and dressing	Issues with self-care	Requirement for care	In nursing home
Cardiovascular disease	Self-reported history of MI	Ischaemic heart disease	Ischaemic heart disease
			Left ventricular dysfunction
		Respiratory disease	Lung disease
Orthostatic hypotension	Orthostatic hypotension	Hypotension/syncope	Orthostatic_hypotension
		Osteoporosis	Osteoporosis
		Parkinsonism and tremor	Parkinsonism and tremor
		Peripheral vascular disease	Peripheral vascular disease
		Polypharmacy	Polypharmacy
		Housebound	Terminal illness
		Thyroid disease	Thyroid disease
Underweight	Underweight	Weight loss and anorexia	Unintentional weight loss
		Urinary system disease	Urinary system disease
		Heart valve disease	Valvular heart disease

Limited physical activity in past 4 weeks	Limited physical activity in past 4 weeks	Activity limitation	
		Dyspnoea	
		Falls	
		Foot problems	
		Hearing impairment	
Difficulty performing moderate activities	Difficulty performing moderate activities	Mobility and transfer problems	
		Peptic ulcer	
		Skin ulcer	
	Trouble sleeping	Sleep disturbance	
Physical or emotional problems interfereing with social activities in past 4 weeks	Physical or emotional problems interfereing with social activities in past 4 weeks	Social vulnerability	
		Urinary incontinence	
		Visual impairment	
	Albuminuria (outside normal range)		
Urea (outside normal range)	Blood Urea Nitrogen (outside normal range)		
Diastolic Blood Pressure (raised)	Diastolic Blood Pressure (raised)		
Difficulty climbing stairs	Difficulty climbing stairs		
	Emotional problems in past 4 weeks		
	Feeling downhearted in past 4 weeks		
	Gait Speed		
Glucose (outside normal range)	Glucose (outside normal range)		
HDL Cholesterol (outside normal range)	HDL Cholesterol (outside normal range)		
	Lacking energy in past 4 weeks		
Overweight	Overweight / Obesity		
Pain interfering with nomal work in past 4 weeks	Pain interfering with nomal work in past 4 weeks		
Potassium (outside normal range)	Potassium (outside normal range)		

	Reduced Digit Symbol - Copy		
	Reduced Logical Memory Delayed Recall		
	Reduced MoCA Orientation Score		
	Self-rated general health		
	Self-reported history of angina		
Smoking status	Smoking status		
Sodium (outside normal range)	Sodium (outside normal range)		
Systolic Blood Pressure (raised)	Systolic Blood Pressure (raised)		
Total Cholesterol (outside normal range)	Total Cholesterol (outside normal range)		
Activities of daily living (ADL) - cannot go out of the house and walk along the road without assistance			
Activities of daily living (ADL) - cannot wash without assistance			
Activities of daily living (ADL) - incontinent of urine			
Creatinine (outside normal range)			
Difficulty bending, kneeling or stooping			
Difficulty carrying groceries			
Difficulty climbing 1 flight of stairs			
Difficulty performing work			
Difficulty walking more than a 100 yards			
Difficulty walking more than a half mile			
Difficulty walking more than a mile			
Geriatric Depression Score (GDS) - Do you feel full of energy?			
Geriatric Depression Score (GDS) - Do you feel you have more problems with memory than most?			
Geriatric Depression Score (GDS) - Do you prefer to stay at home rather than going out and doing new things?			

Geriatric Depression Score (GDS) - Have you dropped many of your activities and interests?			
Haemoglobin (outside normal range)			
Orientation Memory Concentration (OMC) test - Can the patient give the correct time (to within one hour)?			
Orientation Memory Concentration (OMC) test - Can the patient name the month?			
Orientation Memory Concentration (OMC) test - Can the patient name the year?			
Orientation Memory Concentration (OMC) test - Please count backwards from 20 subtracting 1 at a time			
Orientation Memory Concentration (OMC) test - Please repeat the name and address that I told you at the beginning of these questions			
Orientation Memory Concentration (OMC) test - Please say the months of the year backwards			
Patient reported bad taste in mouth within past 4 weeks			
Patient reported blurred vision within past 4 weeks			
Patient reported cold hands or feet within past 4 weeks			
Patient reported constipation within past 4 weeks			
Patient reported heart thumps/misses a beat in past 4 weeks			
Patient reported racing heart within past 4 weeks			
Patient reported shortness of breath within past 4 weeks			
Patient reported swollen ankles within past 4 weeks			
Patient reported weak limbs within past 4 weeks			
Proteinuria (outside normal range)			
Reduced time performing different kinds of work in past 4 weeks			
Reduced time performing nomal work in past 4 weeks			
Severity of bodily pain in past 4 weeks			
Uric Acid (outside normal range)			

Table S3. Characteristics of patients eligible for each trial

Characteristic	Total population		HYVET eligible		SPRINT eligible		OPTiMISE eligible	
	Mean/No.	SD/%	Mean/No.	SD/%	Mean/No.	SD/%	Mean/No.	SD/%
Total population	15,370		268		5290		3940	
**Patient characteristics**								
Age (years)	85.5	4.5	85.6	4.6	85.0	4.3	84.8	4.0
Sex (% female)	9,043	58.8%	180	67.2%	3,255	61.5%	2,285	58.0%
Systolic blood pressure (mmHg)	132.7	15.7	169.3	9.0	141.2	9.7	130.8	11.8
Diastolic blood pressure (mmHg)	72.7	9.6	81.2	10.6	75.6	8.9	71.0	9.0
Body mass index (kg/m^2)	26.4	6.0	26.6	10.4	26.4	5.7	27.7	6.6
Frailty score – fit	6,673	43.3%	136	50.8%	3,341	63.2%	1,222	31.0%
Frailty score – less fit	6,840	44.4%	113	42.2%	1,795	33.9%	2,179	55.3%
Frailty score – frail	1,863	12.1%	19	7.1%	154	2.9%	539	13.7%
Electronic Frailty Index	0.12	0.08	0.10	0.07	0.08	0.06	0.14	0.07
**Medical history**								
Hypertension	9,502	61.8%	176	65.7%	3,161	59.8%	3,522	89.4%
Ischemic heart disease	2,774	18.0%	43	16.0%	609	11.5%	972	24.7%
Myocardial infarction	1,120	7.3%	22	8.2%	235	4.4%	397	10.1%
Stroke/transient ischemic attack	2,244	14.6%	32	11.9%	0	0.0%	514	13.0%
Acute coronary syndrome	149	1.0%	2	0.7%	32	0.6%	60	1.5%
Coronary artery bypass graft	994	6.5%	17	6.3%	216	4.1%	384	9.7%
Heart failure	1,235	8.0%	0	0.0%	216	4.1%	413	10.5%
Peripheral vascular disease	750	4.9%	11	4.1%	194	3.7%	223	5.7%

Cardiovascular disease	5,506	35.8%	75	28.0%	913	17.3%	1,671	42.4%
Atrial fibrillation	2,419	15.7%	27	10.1%	589	11.1%	848	21.5%
Diabetes	2,544	16.5%	39	14.6%	0	0.0%	905	23.0%
Chronic kidney disease	4,343	28.2%	67	25.0%	1,190	22.5%	1,451	36.8%
Cancer	2,880	18.7%	49	18.3%	954	18.0%	746	18.9%
Dementia	1,937	12.6%	0	0.0%	0	0.0%	0	0.0%
Urinary system disease	5,416	35.2%	84	31.3%	1,690	31.9%	1,348	34.2%
Multi-morbidity (2 or more conditions)	11,910	77.5%	201	75.0%	3,446	65.1%	3,542	89.0%
Mean number of morbidities	3.2	2.1	2.9	1.9	2.4	1.7	3.6	1.8
**Prescribed medications**								
Antihypertensive	9,871	64.2%	168	62.7%	3,249	61.4%	3,940	100.0%
Statin	6,486	42.2%	90	33.6%	1,698	32.1%	2,402	61.0%
Antiplatelet	3,909	25.4%	68	25.4%	837	15.8%	1,251	31.8%
Diabetic medication	1,788	11.6%	25	9.3%	12	0.2%	671	17.0%
CVD polypharmacy*	5,477	35.6%	87	32.5%	1,355	25.6%	3,081	78.2%

*Defined as prescription of ≥3 antihypertensives, statins, antiplatelets or diabetic medications; CVD=cardiovascular disease

Figure S1. Sensitivity analysis: Coefficient plot showing results of logistic regression analysis examining predictors of eligibility for each trial (complete cases only)

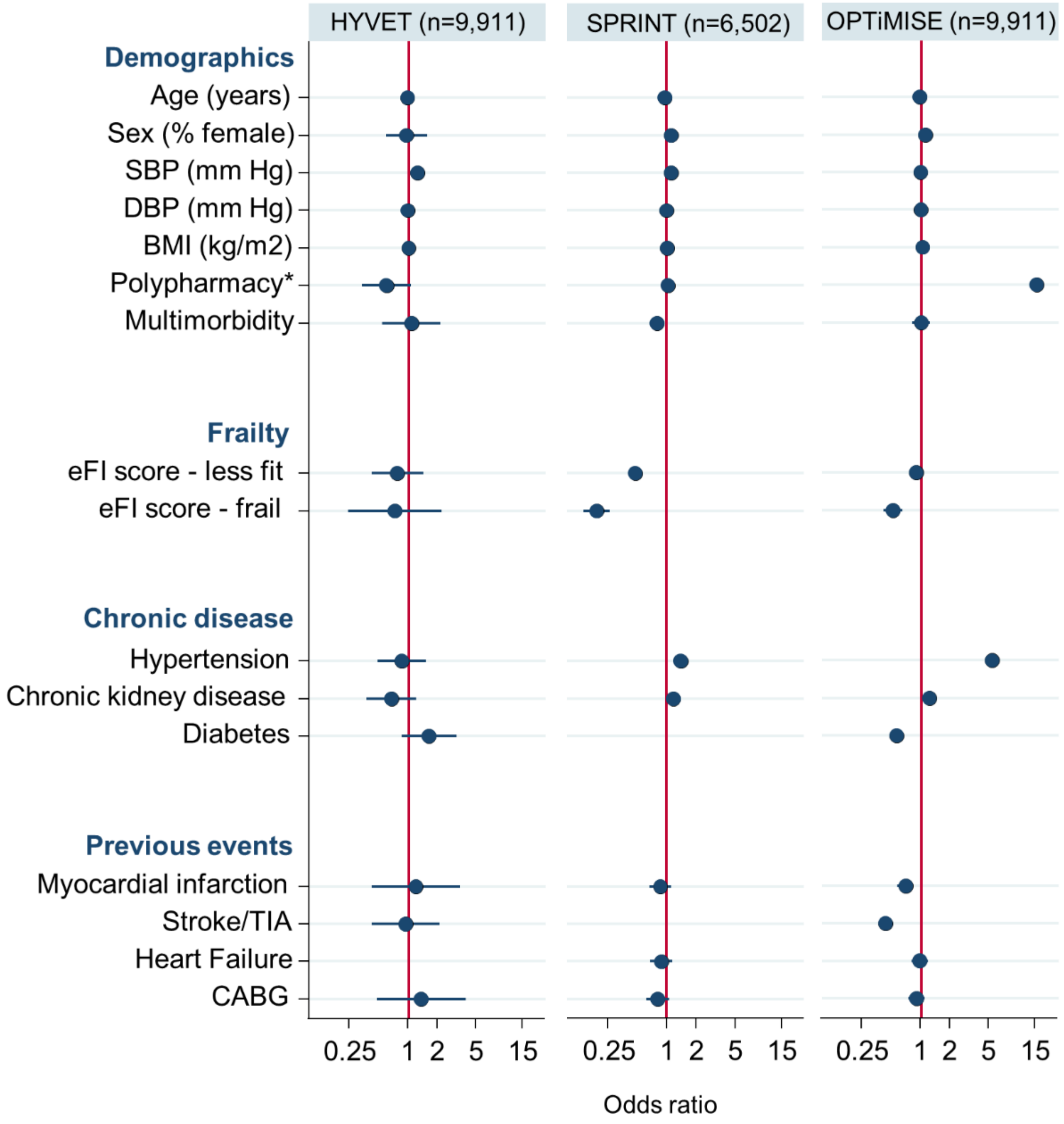

SBP=systolic blood pressure; DBP=diastolic blood pressure; BMI=body mass index; TIA=transient ischemic attack; CABG=coronary artery bypass graft; eFI=electronic frailty index

*Polypharmacy defined as being prescribed 3 or more cardiovascular medications

Figure S2. Sensitivity analysis: Coefficient plot showing results of logistic regression analysis with backwards stepwise selection of candidate predictors of eligibility for each trial (complete cases only)

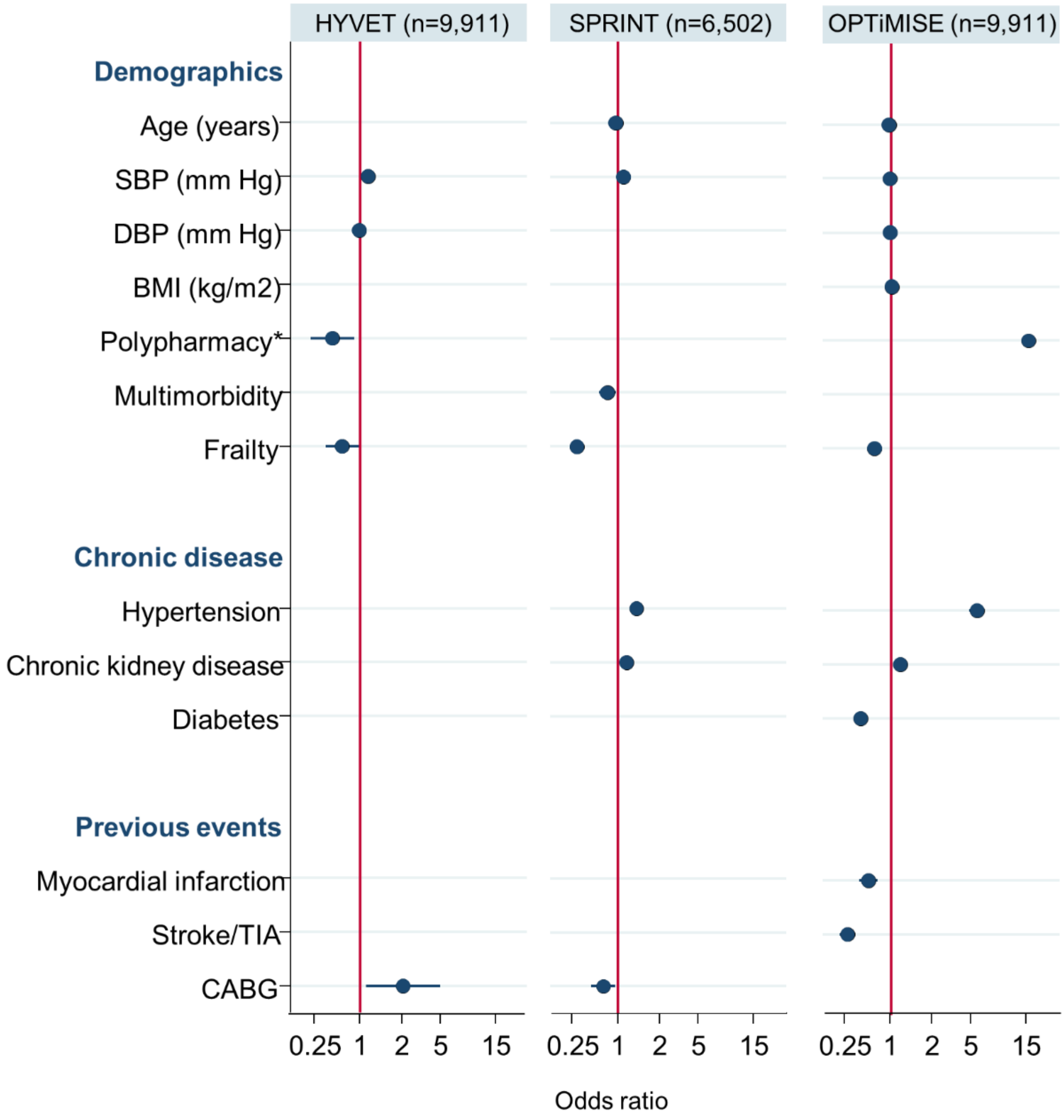

SBP=systolic blood pressure; DBP=diastolic blood pressure; BMI=body mass index; TIA=transient ischemic attack; CABG=coronary artery bypass graft.

*Polypharmacy defined as being prescribed 3 or more cardiovascular medications.
